# Alzheimer’s disease Aβ assemblies mediating rapid disruption of synaptic plasticity and memory

**DOI:** 10.1186/1756-6606-5-25

**Published:** 2012-07-17

**Authors:** Igor Klyubin, William K Cullen, Neng-Wei Hu, Michael J Rowan

**Affiliations:** 1 Department of Pharmacology and Therapeutics, and Trinity College Institute of Neuroscience, Trinity College Dublin, Dublin 2, Ireland

**Keywords:** Amyloidogenic proteins, Long-term potentiation, Long-term depression, Alzheimer’s disease, Neurodegenerative diseases, α-synuclein oligomers, PrP oligomers

## Abstract

Alzheimer’s disease (AD) is characterized by episodic memory impairment that often precedes clinical diagnosis by many years. Probing the mechanisms of such impairment may provide much needed means of diagnosis and therapeutic intervention at an early, pre-dementia, stage. Prior to the onset of significant neurodegeneration, the structural and functional integrity of synapses in mnemonic circuitry is severely compromised in the presence of amyloidosis. This review examines recent evidence evaluating the role of amyloid-ß protein (Aβ) in causing rapid disruption of synaptic plasticity and memory impairment. We evaluate the relative importance of different sizes and conformations of Aβ, including monomer, oligomer, protofibril and fibril. We pay particular attention to recent controversies over the relevance to the pathophysiology of AD of different water soluble Aβ aggregates and the importance of cellular prion protein in mediating their effects. Current data are consistent with the view that both low-n oligomers and larger soluble assemblies present in AD brain, some of them via a direct interaction with cellular prion protein, cause synaptic memory failure. At the two extremes of aggregation, monomers and fibrils appear to act in vivo both as sources and sinks of certain metastable conformations of soluble aggregates that powerfully disrupt synaptic plasticity. The same principle appears to apply to other synaptotoxic amyloidogenic proteins including tau, α-synuclein and prion protein.

## Introduction

Many different amyloidogenic proteins form water insoluble deposits in the brains of patients who die from neurodegenerative diseases [1-3]. The common observation of extensive synaptic loss and mixed neuropathology in many of these diseases suggests that different amyloidogenic proteins may share similar synaptic actions and effects [4-8]. The most frequent cause of neurodegenerative dementia, Alzheimer’s disease (AD), is characterized by profound episodic memory loss which usually presages cognitive decline. The discovery that the hallmark extracellular senile plaques found in the patients’ brains are largely composed of water insoluble fibrillar amyloid ß-protein (Aβ) laid the foundation of the amyloid cascade hypotheses of disease aetiology and led to the investigation of the deleterious effects of Aβ on memory and related neurophysiological processing [9-11].

In the light of the many recent reviews of the cellular mechanisms [12-16], the present review focuses on defining the roles of different Aβ assemblies [17,18] in Aβ-mediated synaptic and memory disruption. Since cognitive status in patients with AD is much more strongly correlated with brain concentration of water soluble Aβ rather than insoluble fibrillar Aβ-containing plaque load [19,20], most recent research has focused on soluble species of Aβ.

**Figure 1:**
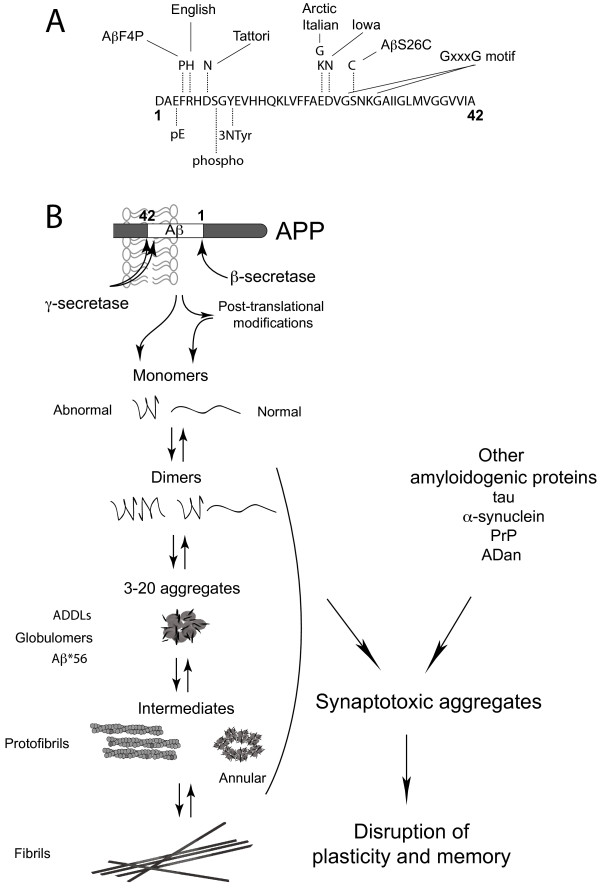
**Schematic representation of Aβ processing and aggregation. **(**A**) Primary sequence of human Aß1-42 with examples of natural or designed intra-Aß mutations (above sequence) and post-translational modifications (below sequence). (**B**) Amyloid precursor protein (APP) cleavage by β- and γ- secretases releases aggregation-prone Aβ peptides, particularly Aβ1-42. Intra-Aβ mutations and post-translational modifications increase Aβ ability to aggregate even more. It has been suggested that diffusible Aβ aggregates rather than monomer form or fibrils are the synaptotoxic species. These aggregates include ADDLS (Aß-derived diffusible ligands), globulomers (globule-like 12-mers), Aß*56 (56 kDa Aß-containing aggregates derived from brain), protofibrils (soluble, short fibril-shaped often “worm-like” structures) and annular protofibrils (protofibrils that can form pores in membranes). Other aggregation-prone proteins also form synaptotoxic soluble species that may share conformation recognized by antibodies.

In order to investigate the effects of different Aβ species on memory and related synaptic mechanisms, acute treatment with Aβ provides a relatively simple but very attractive and manipulable model system, compared to transgenic amyloid precursor protein (APP) animal models [21]. The acute application approach gives the opportunity to control and characterize the biophysical state of aggregation-prone Aβ preparations prior to use. Since pioneering *in vivo* studies found that injection of synthetic Aβ-related peptides of undefined assembly can impair learning [22,23] and reduce synaptic transmission in the hippocampus of the rat brain [24], this approach has been exploited in order to examine the role of different Aβ assemblies. By comparing the relative activity of different soluble preparations of Aβ in these acute models it is hoped that it will be possible to determine the nature and actions of synaptic and memory disrupting assemblies. These assemblies vary in primary sequence, size and putative generic conformation. They include monomers, low-n oligomers, larger oligomers such as Aβ derived diffusible ligands (ADDLs) [25,26] and globulomers [27], and protofibrils which are usually shorter and thinner than insoluble amyloid fibrils [28] (Figure 1). Currently there is little agreement as to which, if any, of these assemblies is most culpable in causing synaptic plasticity and memory disruption. The present review examines recent evidence, including the actions of other amyloidogenic peptides and the possible involvement of cellular prion protein (PrP^C^) as a selective target of certain oligomers.

## Acute synaptic and behavioural effects of Aβ

Two of the most sensitive and robust measures of the acute synaptic disruptive effects of Aβ are inhibition of long-term potentiation (LTP) [29] and facilitation of long-term depression (LTD) of excitatory synaptic transmission [30], both of which engage plasticity mechanisms believed to underlie certain types of learning and memory [31-33]. Baseline synaptic efficacy appears more resistant to the effects of Aβ in most acute studies. Some of the most sensitive behavioural indicants of rapid impairment of cognition and memory include performance of operant tasks [34] and aversive learning [35].

## Aβ amino acid sequence and post-translational modification

The cleavage of APP by the γ-secretase complex is permissive, with Aβ1-40 the dominant Aß species (Figure 1) [17]. In AD brain the concentrations of highly amyloidogenic species, especially the more potent synaptic plasticity-disrupting Aβ1-42 [29,36], increase. Since the discovery of rare early-onset autosomal dominant familial forms of AD caused by missense mutations of the APP gene within the Aβ region, synthetic peptides bearing familial and design mutations have been used to investigate the potential importance of primary sequence in determining Aβ aggregation, toxicity and synaptic disruption [37]. Some years ago we found that Arctic synthetic mutant Aβ1-40(E22G) peptide, which has a much greater tendency than Aβ1-40 to form soluble aggregates including protofibrils, is accompanied by a greater potency to block LTP [38]. More recently Tomiyama et al. [39] reported that familial AD-associated Aβ that lacks glutamate-22 showed enhanced oligomerization in the apparent absence of fibril formation, and was a more potent inhibitor of LTP.

Beyond the primary sequence, biochemical modifications of Aβ, including post-translational processing, can lead to the generation of highly aggregation prone species in the brain [40,41]. Aminopeptidase removal of residues 1 and 2 of Aß followed by glutaminyl cylase-mediated cyclization of the exposed glutamate to a pyroglutamate, leads to the production of N-terminally truncated pyroglutamate –modified variants of Aβ (Aβ3pE-4x) [42] (Figure 1) which have been proposed to be particularly pathogenic [43]. In agreement, Aβ3pE-42 impairs spatial working memory and retention of reference memory in mice after intracerebroventricular (i.c.v.) injection with a similar potency to Aβ1-42 [44]. In a detailed structure-activity relationship analysis, freshly prepared synthetic Aβ3pE-x inhibited LTP *in vitro* with the following order of potency: Aβ3pE-42 > Aβ1-42 = Aβ3pE-38 = Aβ3pE-40 >> Aβ1-40, Aβ1-38 or Aβ3-40, the latter three being inactive at the highest concentration tested [45]. The authors found that this activity correlated with the relative ability to rapidly form oligomers and short fibrillar aggregates. Clearly the N-terminus of Aβ can play a critical role in determining aggregation and hence, presumably, ability to disrupt synaptic plasticity.

**Figure 2:**
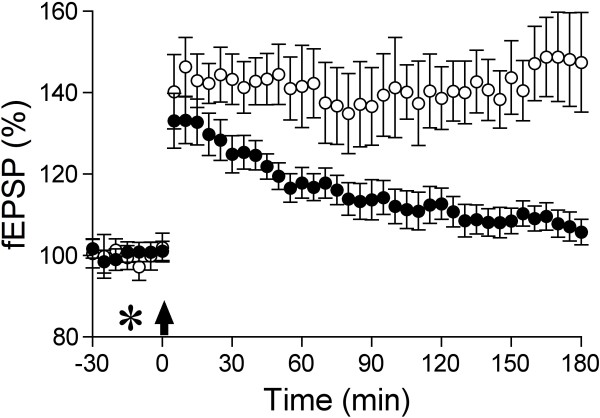
**Aβ1-42 oligomers prepared from a precursor isopeptide by direct dissolution in physiological buffer **[50 ]**inhibits long-term potentiation in the hippocampus *in vivo*. **Animals were injected with either vehicle (open circles) or Aβ1-42 (2.5 μg, closed circles) by intracerbroventricular injection (asterisk) 10 min before the conditioning high frequency stimulation (HFS, arrow) in the CA1 area of the anaesthetized rat hippocampus. Values are the mean ± SEM baseline field EPSP (fEPSP) (n = 6-7 per group).

Somewhat similarly, nitration of Aß at tyrosine 10 also promotes aggregation and increases the magnitude of inhibition of LTP by Aß1-42 in hippocampal slices [46]. Such nitration is likely to arise subsequent to the formation of secondary products of NO production by pro-inflammatory upregulation of inducible nitric oxide synthase and can be pharmacologically targeted [46]. It will be interesting in future studies to determine if the activity in acute synaptic plasticity and memory models of other Aβ species found in AD brain, including Aβx-43 [47], phosphorylated Aβ [48] and glycosylated Aβ [49] relates to their tendency to form specific aggregates. One of the difficulties of working with aggregation prone peptides is to ensure consistent starting material in the absence of extensive solvent pretreatment. Recently, it has been shown that Aβ1-42 aggregates can be reliably generated from a precursor isopeptide by direct dissolution in physiological buffers [50]. Aβ oligomers prepared in this manner impede spatial learning and inhibit LTP both *in vitro*[50] and *in vivo* (Klyubin et al., unpublished) (Figure 2).

## Aβ assembly size

### Aβ monomers

What do we know about the synaptic and mnemonic activity of Aβ monomers? Despite the natural ability of Aβ, especially the human sequence, to form aggregates, the majority of Aβ prepared by chemical synthesis or Aβ produced naturally by cells *in vitro* and the brain *in vivo* usually contains a sizable fraction of Aβ monomers. Biophysical methods such as size exclusion chromatography (SEC) are employed to enrich them. The results obtained by our group and by others suggest that Aβ monomers probably have little or no ability to disrupt synaptic functioning. Firstly, Aβ monomers produced by cultured CHO cells overexpressing human APP, known as 7PA2 cell line, did not affect LTP *in vivo*[51] or learned behavior [34]. Secondly, SEC fractions of Aβ monomers from AD brain homogenates and native human cerebrospinal fluid (CSF) failed to inhibit LTP *in vitro* and *in vivo*, respectively [35,52]. Thirdly, SEC-separated monomers of a synthetic analog of Aβ1-40, Aβ1-40(S26C), had no effect on LTP in the CA1 area *in vivo*[53]. Fourthly, and most recently, Aβ 1-42 monomers, used in the preparation of oligomers using photo-induced cross-linking of unmodified proteins (PICUP), failed to inhibit LTP or facilitate LTD induction in hippocampal slices [54].

Some “physiological” effects of Aß on synaptic transmission, plasticity and learning have been described [55-59]. For example, extremely low concentrations of Aβ, both exogenously applied and endogenously generated, can enhance synaptic LTP and improve performance of learning tasks [58]. Because neurotrophic and neuroprotective effects of Aβ in cultured cells [60] have been attributed to Aβ monomers [61], it might be expected that these apparently positive effects are mediated by Aβ monomers [58,59]. However, Puzzo et al. [58] reported that whereas pre-aggregated Aβ1-42 reversed the impairment of LTP caused by an anti-rodent Aβ antibody, they did not detect any effect of a synthetic Aβ1-42 preparation enriched in monomers. It will be of interest to determine if the same applies to the more abundant Aβ1-40 or cell-derived Aβ.

### Small and large aggregates

Given the findings that Aβ monomers *per se* don't appear to impair synaptic function, the question arises as to which soluble Aβ aggregates are disruptive. Several lines of evidence suggest that small highly diffusible Aβ aggregates may be responsible for memory impairment in AD [2,62]. The size of these aggregates varies from Aβ dimers, containing only two Aβ molecules, to approximately 20-mers. Although several protocols for the generation of well-characterized synthetic Aβ aggregates have been established to date, the question remains as to how relevant these Aβ assemblies are to the situation in an AD brain *in vivo* where there is a complex mixture of potentially interacting species [17,18,63].

Acute administration of extremely low doses of low-n Aβ oligomer-enriched fractions of conditioned medium from cultured 7PA2 cells rapidly disrupts synaptic plasticity [51] and performance of learned behaviours [34,64]. In contrast, medium from APP transfected HEK293 cells that contained Aβ1-x or Aβ3-x peptides as a mixture of monomers and dimers (total Aβ concentration ~700 nM) did not significantly inhibit LTP [45]. However medium containing soluble large oligomers of Aβ3pE-x peptides in addition to an equivalent amount of monomer/dimer, inhibited LTP, indicating that larger assemblies of some natural Aβ may be particularly active.

Interestingly, the impairment of avoidance learning by 7PA2 conditioned medium, that contains low-n Aβ oligomers but no detectible large soluble assemblies such as protofibrils, is associated with disruption of synaptic remodeling in the dentate gyrus [65]. Furthermore, recall of hippocampus-dependent contextual fear learning is more susceptible to impairment than recall of amygdala-dependent cued learning after i.c.v. injection of 7PA2 conditioned medium [66]. These studies indicate that low-n oligomers may have preferential interactions with synapses in key hippocampal pathways.

**Figure 3:**
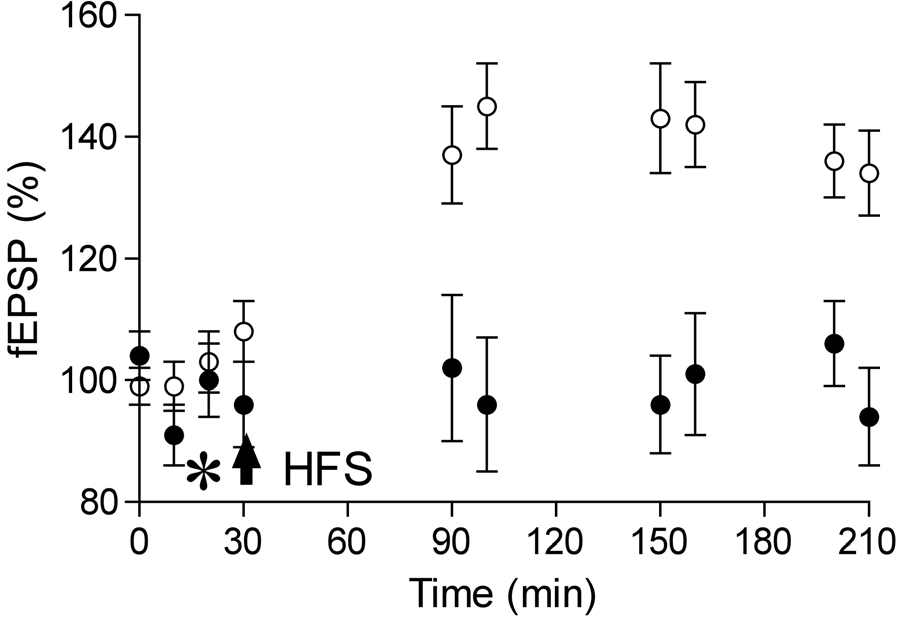
**Aβ in soluble extracts of Alzheimer’s disease brain inhibits LTP *in vivo*. **Representative example of LTP impairment after intracerebroventricular (i.c.v.) injection (asterisk) of Tris-buffered saline (TBS) extract of an AD brain containing Aβ (130 pg in total) (closed circles). In vehicle-injected controls, HFS (arrow) induces stable LTP (open circles) in the CA1 area of the anaesthetized rat.

The group led by Selkoe, having shown the presence of various Aβ assemblies in AD brain, suggested that soluble Aβ dimers are the smallest synaptotoxic species [35]. Indeed, a combination of biochemical analysis, electrophysiological experiments and behavioral tests revealed that sodium dodecyl sulfate (SDS) stable Aβ dimers found in water soluble extracts of AD brain disrupt the performance of an aversive learning task, inhibit LTP and facilitate LTD in rodents [35,67-69]. AD brain soluble Aβ, containing SDS-stable dimers, disrupts synaptic plasticity in a dose-dependent manner and is very potent (Klyubin et al., unpublished observations) (Figure 3). In contrast, the larger Aβ*56 oligomers extracted from APP transgenic mouse brain [70] appear to be much less potent than cell-derived low-n oligomers or human brain Aβ dimer-containing soluble extracts at causing deficits in cognitive tasks [35,64].

In apparent support of the proposal that dimers are key synaptotoxic species, synthetic Aβ1-40 dimers blocked LTP both *in vivo* and *in vitro* when acutely applied at a concentration approximately 50-fold lower than unmodified Aβ1-40 [35,53]. These dimers were created using Aβ with a single conservative amino acid substitution (cysteine in place of serine 26, S26C) enabling covalent cross-linking with a disulfide bond under oxidizing conditions. However, soon after this, Walsh and colleagues found evidence that these dimers need to assemble into large protofibril-like aggregates before being able to potently inhibit LTP [71]. In fact, freshly prepared non-aggregated synthetic Aβ1-40(S26C) dimers, like monomers (see above), were found to have no significant effect on synaptic plasticity whereas protofibril-rich assemblies of these dimers strongly inhibited LTP *in vitro*. An explanation given by the authors for the discrepancy from the earlier findings was a lack of definition of aggregation state of materials used in previous studies. This conclusion is in agreement with the work of another group who demonstrated that tissue transglutaminase, an enzyme implicated in neurodegeneration with the catalytic capability to covalently cross-link “wild type” Aβ between lysine and glutamine residues, induced synthetic Aβ1-40 to form large assemblies including protofibrils which potently inhibited LTP in the CA1 area *in vitro*[72]. In contrast, a similar low concentration (100 nM) of untreated Aβ1-40 had no effect.

Some synthetic Aβ low-n and high-n oligomers are not harmful to neurons. Thus aggregation of synthetic Aß1-42 where the glycine residue at position 33 is substituted with alanine generated Aβ1-42(G33A) tetramers which failed to inhibit LTP, as was the case with Aβ1-42(G33I) which only formed high-n oligomers when aggregated [73].

Just as in the case of the disruptive effects of synthetic Aβ on synaptic plasticity, there is evidence that only certain “intermediate” synthetic Aβ assemblies, including protofibrils, can rapidly impair learning [74,75]. However, regardless of the relationship between size of soluble Aβ aggregates and synaptic dysfunction, insoluble fibrils per se are unlikely candidates for memory impairment in AD. Rather, plaque-containing insoluble fibrils are likely to provide a major source and sink of memory disrupting soluble Aβ [35,74].

Because of difficulties in determining the size of biologically active Aβ aggregates, especially under non-denaturing conditions, size-selective ligands such as antibodies should prove useful. Recently, O'Nuallain et al. [76] developed an antibody, 3C6, that preferentially binds soluble aggregates of covalently cross-linked dimers of Aβ1-40(S26C), and recognizes only a portion of SDS-stable dimers in aqueous extracts of AD brain [76]. Importantly, such apparent selectivity was sufficient to prevent block of LTP by the AD brain soluble extract *in vivo*. It is possible that 3C6-mediated abrogation of LTP inhibition triggered by AD brain soluble Aβ was due to rapid direct neutralization of aggregates of Aβ larger than single SDS-stable dimers.

In an analogous approach with synthetic Aβ, N7, an agent believed to selectively block large Aβ assemblies that form ion-permeable pores in membranes, prevented Aβ aggregate-induced depletion of presynaptic glutamatergic vesicles and consequent depression of spontaneous synaptic currents in cultured hippocampal neurons [77].

## Conformation versus size

Not only size, but also the spatial conformation of synapse-disruptive soluble Aβ aggregates varies. Thus Aβ aggregates can be classified based on the ability of conformation-specific antibodies to recognize aggregates in a manner that appears relatively independent of size [78]. Such conformation-specific antibodies, for example, are used to distinguish between so-called “prefibrillar” and “fibrillar” types of aggregates regardless of their size. Thus, ADDLs and globulomers are likely to be “fibril”-type whereas Aβ*56 is probably “prefibrillar”. As a corollary to the ability of different sized aggregates to adopt similar conformations, the same sized Aβ aggregates may have different sub-populations of different conformers.

Evidence suggestive of a relatively “size-independent” role for an N-terminal ß strand conformation in the synaptic plasticity disrupting effects of synthetic Aß oligomers and protofibrils has been reported [79]. Thus, synthetic Aß1-40, containing oligomers and protofibrils in the presence of a ß-sheet breaker peptide corresponding to residues 4-10 of Aß, designed to reduce the relative amount of N-terminal ß strand conformation, failed to inhibit LTP. In contrast, synthetic Aß1-40 containing a point mutation (P4F) that promoted the formation of protofibrils, including those with an N-terminal ß-strand conformation, inhibited LTP *in vitro* with a similar potency to an oligomer preparation of wild type Aß1-40 with a similar ß-strand conformation.

Like Aβ, many other amyloidogenic proteins form aqueous soluble oligomers that are neurotoxic [2,80]. Intriguingly, many of these neurotoxic oligomers adopt similar conformations to Aβ recognized by conformation-selectivez antibodies [78,81,82]. The conformations adopted are relatively independent of their primary amino acid sequence, as is the case for fibrils [83]. For example, the antibody A11, originally generated against Aβ oligomers, recognizes a common conformation adopted by oligomers of many peptides, including α−synuclein and an amyloidogenic fragment of PrP^C^, PrP106-126 [84,85]. Whether or not conformational epitopes on Aβ and other peptide aggregates determine their ability to selectively bind to specific synaptic sites and thereby disrupt memory mechanisms has yet to be resolved but there is growing suggestive evidence consistent with the hypothesis, as outlined below.

Soluble oligomers of tau, the main protein deposited intracellularly as insoluble fibrils in AD and fronto-temporal dementia, can rapidly impair object recognition memory and reduce levels of synaptic vesicle-associated proteins when applied intrahippocampally *in vivo*[86]. In contrast, monomers and fibrils of tau appeared inactive under the same acute treatment protocol.

Insoluble aggregates of α−synuclein are the main constituent of intracellular inclusions, Lewy bodies, in the brains of patients with Parkinson’s disease and related dementias, but soluble oligomers are released extracellularly and are neurotoxic [87]. Intriguingly, low nanomolar concentrations of large α−synuclein oligomers can rapidly trigger a selective increase in AMPA receptor-mediated synaptic transmission in autaptic neuronal cultures [88]. In contrast, other oligomers of α−synuclein were reported to inhibit LTP without affecting baseline transmission, and to impair learning an avoidance task [89].

**Figure 4:**
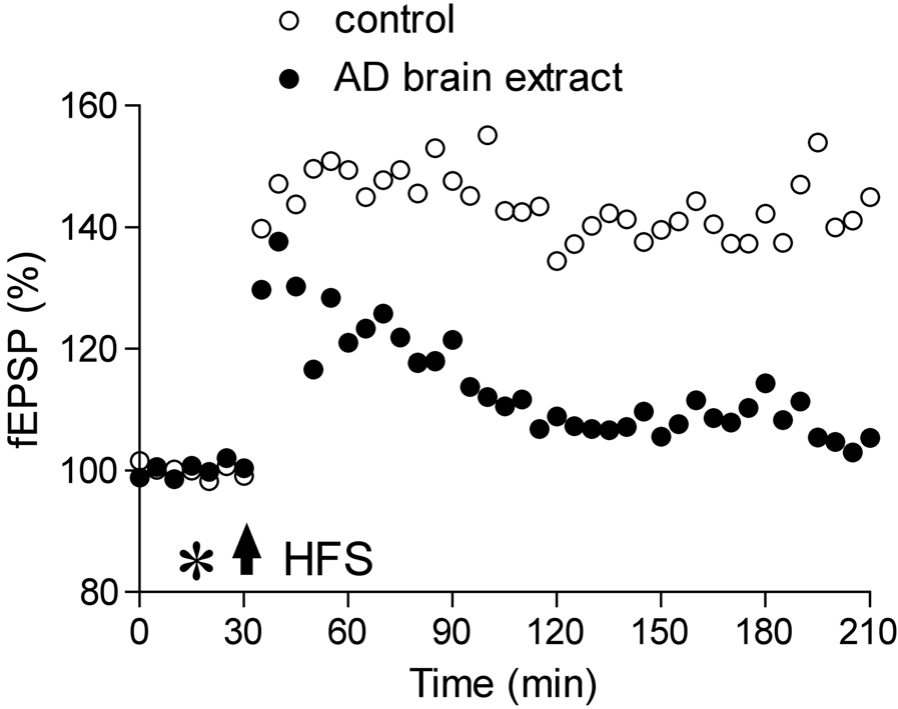
**The aggregation-prone prion protein fragment PrP106-126 inhibits LTP *in vivo*. **Animals were injected with either vehicle (open circles) or PrP106-126 (96 ng, closed circles) by i.c.v. injection (asterisk) 10 min before the HFS (arrow) in the CA1 area of the anaesthetized rat hippocampus. Values are the mean ± SEM baseline fEPSP (n = 10 per group).

The prion peptide fragment PrP106-126 is used to model the neurotoxic, rather than the infective, aspects of prion-mediated transmissible spongiform encephalopathies (TSEs)[90-95]. In prion diseases synaptic mechanisms are often disrupted at a relatively early stage [96]. Intriguingly, i.c.vinjection of PrP106-126 inhibits LTP of synaptic transmission in the CA1 area of the hippocampus *in vivo* (Cullen et al., unpublished observations) (Figure 4).

Another amyloidogenic peptide, ADan, is found deposited in the brains of patients with familial Danish dementia, a rare autosomal dominant form of cognitive impairment with AD-like neuropathology. The N-terminally truncated pyroglutamate form of ADan was found to be especially prone to aggregate into large oligomers and appeared to be more potent than unmodified ADan at inhibiting LTP *in vitro*[45].

In view of the shared ability of conformational antibodies to recognize these aggregates and their shared ability to inhibit LTP it is tempting to speculate that a common conformation is critical for the synaptic plasticity and hence memory disrupting actions of these very different peptides. In line with this and similar to the situation with regards the role of Aβ aggregate size, future studies should attempt to resolve which, if any, specific conformation of soluble Aβ assemblies is more disruptive to synapses and memory.

## Cellular prion protein and Aβ-mediated disruption of synaptic plasticity and learning

Given the likely key pathogenic role of a partially protease-resistant misfolded form of PrP^C^ (PrP^Sc^), and the critical requirement for PrP^C^, in transmissible spongiform encephalopathies [8,97], the relationship and commonalities between prion-mediated neurodegenerative diseases and AD have become a major focus of research [98-104].

Recently synthetic Aβ oligomer-mediated inhibition of LTP at hippocampal synapses *in vitro* was reported to be dependent on PrP^C^[105] with Aβ oligomers, but not monomers or fibrils, potently and selectively binding specific regions of PrP^C^, especially in the vicinity of amino acids 95-105 [105-107]. Antibodies that bind PrP^C^ within the region of 93-109 [105] or 93-102 [107] prevented the inhibition of hippocampal LTP by synthetic Aβ1-42 oligomers *in vitro*. Consistent with these reports, the *in vivo* synaptic plasticity disrupting actions of AD brain extracts containing water soluble Aβ were dependent on PrP^C^[67]. Thus, the disruptive effect of Aβ was abrogated by D13, an antigen recognizing antibody fragment (Fab) that binds selectively to PrP_96-104_^C^. It is likely that these antibodies and related agents are directly obstructing the binding of Aβ oligomers to PrP^C^. In addition, an antibody to the alpha helix of PrP^C^ also prevented the inhibition of LTP by AD brain Aβ oligomers both *in vitro* and *in vivo*[107] whereas a Fab directed to the C-terminus of PrP^C^ appeared to be inactive [67]. Since the alpha helix of PrP^C^ does not overlap with the putative binding site of Aβ oligomers, one possible explanation for these findings is that the antibody to the alpha helix is interfering with PrP:PrP contact. Interestingly, direct intra-hippocampal injection of bivalent D13 antibodies, but not monovalent D13 Fabs, can cause delayed apoptotic neurodegeneration in mice [108], but see [109], indicating that abnormal cross-linking of PrP^C^ in the 96-106 region by oligomers may contribute to their damaging effects. Indeed, cross-linking of PrP^C^ has been associated with synaptic damage caused by cell-derived low-n oligomers of Aβ in cultured neurons [110]. Such cross-linking Aβ oligomers may prevent PrP-dependent inactivation of N-methyl-d-aspartate (NMDA) receptor-mediated currents leading to abnormally enhanced NMDA receptor-mediated glutamatergic transmission [111]. Furthermore, cross-linking of other adjacent membrane proteins, in particular metabotropic glutamate receptor 5, may go hand-in-hand with this process in mediating Aβ oligomer-induced synaptotoxicity [112].

In apparent direct contradiction to the findings of Lauren et al. and Freir et al. [67,105,107], Kessels et al. [113] reported that Aβ1-42 oligomers impaired LTP in hippocampal slices from transgenic mice lacking PrP^C^. Moreover, in APP transgenic mice a deficit in LTP was similar in the presence or absence of PrP^C^[114]. Differences in the Aβ oligomer concentration/assembly are likely to explain these apparently contradictory findings [107]. In the Kessels et al. study [113], in contrast to most other reports on acute effects of Aβ oligomers on synaptic plasticity, the inhibition of LTP was accompanied by a marked rapid reduction in baseline synaptic transmission. This indicates that concentrations of certain Aβ oligomer-containing preparations sufficient to rapidly reduce baseline transmission can bypass a requirement for PrP^C^ to disrupt synaptic function.

At the behavioural level, there is also strong evidence that Aβ-mediated memory impairment is PrP^C^-dependent [115,116]. However, an apparent acute disruption of object recognition memory caused by Aβ1-42 was not prevented in mice lacking PrP^C^[117]. Moreover, Cissé et al [118] in a recent paper observed the same cognitive deficits in APP transgenic mice in the presence or absence of PrP^C^. These authors provided strong evidence, instead, that impairments of synaptic plasticity and memory were due to a direct interaction of Aβ oligomers with the Ephrin B2 receptor EphB2 [119]. It will be important to determine if the fact that different APP transgenic mice at different ages express different potentially synaptotoxic Aβ assemblies [18,120] can help explain this controversy.

## Conclusion

The commonalities and differences between amyloidogenic proteins in different neurodegenerative diseases are of great theoretical and practical interest. The ability of certain assemblies of these proteins to rapidly disrupt synaptic plasticity and memory mechanisms indicates that there may be shared mechanisms across diseases. An obvious limitation of the acute application approach is that although it is now feasible to apply relatively homogenous protein assemblies, it is not clear how these relatively labile preparations behave structurally throughout the full duration of the experiments and how this may depend on the existing milieu of endogenous amyloidogenic proteins which is known to depend on the ongoing neuronal activity amongst many factors [100,121]. In the light of the chronic nature of these diseases this may prove a difficult but important question to address. Furthermore, the question remains as to how well exogenously applied proteins, especially synthetic aggregates, in rodents, mimic the actions and effects of endogenously generated proteins *in situ* in the brains of patients. To date, the evidence for the involvement of different sizes of aggregates and different cellular targets in these models is compelling. If the same conclusion applies to patients, perhaps with different assemblies playing a leading role at different stages of disease, it probably will be necessary to take this diversity into account when developing new diagnostic and therapeutic approaches. On the other hand, if common conformations of different proteins are pathophysiologically relevant, selectively neutralizing them [51,122], or changing their aggregation kinetics such that monomers are stabilized [54] or even by accelerating their conversion to fibrillar material [123], may have utility in a wide spectrum of neurodegenerative disorders.

## Consent

Each brain donor consented to have their post-mortem tissue used for research.

## Competing interests

The authors declare that they have no conflict of interest to disclose.

## Authors’ contributions

Both IK and MJR drafted and edited the manuscript. IK, NWH and WKC provided their experimental data. All authors read and approved the final manuscript.
